# Short-Term, Low-Volume Training Improves Heat Acclimatization in an Operational Context

**DOI:** 10.3389/fphys.2017.00419

**Published:** 2017-06-16

**Authors:** Keyne Charlot, Pierre-Emmanuel Tardo-Dino, Jean-François Buchet, Nathalie Koulmann, Stéphanie Bourdon, Benoit Lepetit, Martin Roslonski, Loïc Jousseaume, Alexandra Malgoyre

**Affiliations:** ^1^Département Environnements Opérationnels, Unité de Physiologie des Exercices et Activités en Conditions Extrêmes, Institut de Recherche Biomédicale des ArméesBretigny-Sur-Orge, France; ^2^Ecole du Val de GrâceParis, France; ^3^Forces Françaises aux Émirats Arabes Unis, Centre Médical InterarméesZayed Military City, United Arab Emirates

**Keywords:** heat acclimation, heat acclimatization, exercise, training, military, rectal temperature, sweat rate

## Abstract

Personnel who travel to areas with a hot climate (WBGT > 27°C) may suffer from the heat (physiological strain, thermal discomfort, increased probability of heat illness), making them partially or fully inoperative. Performing physical activities during heat acclimatization is known to improve this process (i.e., improve measures of acclimatization for the same duration of acclimation). However, it is unknown whether such training would be efficient in an operative context, characterized by a high volume of work-related physical activity. Thirty French soldiers (Training group, T) performed a short (5 days), progressive, moderate (from three to five 8-min running sets at 50% of the speed at VO_2max_ for 32–56 min) aerobic training program upon arriving at their base in United Arab Emirates (~40°C and 12% RH). A control group (30 soldiers; No Training, NT) continued to perform their usual outdoor military activities (~6 h.d^−1^). A field heat stress test (HST; three 8-min running sets at 50% of the speed at VO_2max_) was performed, before and after the heat acclimatization period, to assess physiological and subjective changes. Rectal temperature, heart rate (HR), thermal discomfort at rest and at the end of exercise, rates of perceived exertion (RPE), and sweat loss and osmolality decreased following heat acclimatization in both groups. However, the decreases in the T group were larger than those in the NT group for HR at the end of exercise (−20 ± 13 vs. −13 ± 6 bpm, respectively, *p* = 0.044), thermal discomfort at rest (−2.6 ± 2.7 vs. −1.4 ± 2.1 cm, respectively, *p* = 0.013) and at the end of exercise (−2.6 ± 1.9 vs. −1.6 ± 1.7 cm, respectively, *p* = 0.037) and RPE (−2.3 ± 1.8 vs. −1.3 ± 1.7, respectively, *p* = 0.035). Thus, we showed that adding short (<60 min), daily, moderate-intensity training sessions during a professional mission in a hot and dry environment accelerated several heat-acclimatization-induced changes at rest and during exercise in only 5 days.

## Introduction

Various professionals perform mid to long-term missions (from 1 week to several months) abroad in areas with a hot climate: soldiers, engineers, and humanitarians, among others. During these stays, they must often be rapidly operational, despite substantial heat stress that can be induced by clothing that impedes heat loss (battledress), the physical work rate (endogenous heat production), and most of all, the environmental conditions (temperature, humidity, or solar radiation) in these areas. These conditions increase physiological strain, reducing physical (Périard et al., 2015; Székely et al., 2015), cognitive, and psychomotor performance (Cian et al., 2000; Hancock et al., 2007), and increasing the occurrence of exertional heat illnesses, such as exertional heat stroke (Pryor et al., 2015), diseases that are common in worker populations (Marchetti et al., 2016) and especially soldiers (Armed Forces Health Surveillance Bureau, 2017). This problem was recognized more than 200 years ago when Europeans arriving in tropical climates did not adapt their working behaviors and eventually faced heat illnesses (Lind, 1771; Jackson, 1795; Taylor, 2014). Heat acclimation (or more accurately heat acclimatization as heat stress is produced by the environment and not simulated in thermal rooms) alleviates these losses in performance by producing physiological changes that reduce the deleterious effects of heat stress (Périard et al., 2015; Tyler et al., 2016). For more details, Taylor (2014) accurately reports the functional modifications that occur during heat acclimation.

Physical activity rates during the acclimatization period highly alter heat acclimation kinetics. Indeed, passive heat acclimatization is less efficient than active heat acclimatization by work-induced physical activity (Horvath and Shelley, 1946; Hellon et al., 1956; Joy et al., 1964; Pandolf et al., 1977) or training sessions (Périard et al., 2015, 2016; Racinais et al., 2015; Tyler et al., 2016). To be effective, in terms of heat acclimation-induced physiological modifications, an exercise session performed in the heat should trigger high sweat rates and largely increase core body temperature (Henane et al., 1977; Racinais et al., 2015). This could be achieved with any session modalities in duration or intensity (Houmard et al., 1990; Gibson et al., 2015), although it is suggested that high-intensity exercise may be more efficient than lower-intensity exercise (Houmard et al., 1990). Training would therefore be equivalent to supplemental heat acclimatization, compared to work-induced heat acclimation alone. Upon arriving in a hot area, personnel are generally requested to spend several hours each day performing light to moderate tasks related to their work. They should therefore rapidly benefit from active heat acclimatization, but it is likely that these tasks are insufficient to produce an optimal effect. This quest for complete heat acclimation to ensure worker efficiency and safety goes back to the beginning of the twentieth century when candidates to work in the gold mines in South Africa were asked to be acclimatized before being enrolled (Dreosti, 1935, 1949; Schneider, 2016). A few studies suggest that adding training sessions to the schedule of professionals with no outdoor tasks may improve physiological changes and thermal discomfort (Aoyagi et al., 1994, 1998; Yamazaki, 2013). However, there is only sparse evidence of the positive effects of physical training during heat acclimatization in an operational context. There is a need therefore to more clearly determine whether training sessions can elicit such positive modifications for professionals for whom work-related tasks are already physically stressful.

The aim of this experiment was to assess the effects of additional moderate, progressive training during short-term heat acclimatization (5 days) in soldiers deployed in the Middle-East during a very hot and dry spring period (more than 40°C and 12% relative humidity) on classical markers of heat acclimation (Sawka et al., 2011), [rectal temperature, heart rate (HR), sweat loss, and sweat osmolality] and thermal discomfort at rest and during moderate exercise performed in dry heat. The originality of this experiment was that all subjects, including the control group (No Training), were highly active in performing outdoor military tasks during the heat acclimatization period. We hypothesized that even a short period of low volume training, adapted to military time-constraints, would improve heat acclimatization and facilitate the realization of military tasks and physical activity. We chose a short period (5 days) because soldiers or personnel in this context were not available for a longer time and were engaged in their tasks by the end of the first week. This period is also sufficient to induce partial or complete modification of several physiological factors (decreased HR and core temperature at rest and during exercise, increased plasma volume) and improve thermal discomfort (Périard et al., 2015; Racinais et al., 2015).

## Methods

### Experimental approach to the problem

A repeated-measures and within-participants design was used to determine whether a short period of low-volume training is sufficient to enhance heat acclimatization in French Army soldiers deployed in desert-like conditions near Abu Dhabi in United Arab Emirates. Physiological factors (HR, rectal temperature, sweat loss, and osmolality) were measured and subjective factors [thermal discomfort using visual analogic scales and rates of perceived exertion (RPE)] were assessed during a heat stress test (HST) performed before and after 5 days of heat acclimatization. One half (*n* = 30) of participants trained, whereas the other half did not participate in this training program. However, all participants performed similar indoor and outdoor physical military activities during this period. Thus, only the effect of training was assessed in this protocol.

### Participants

Sixty French Army soldiers were selected to participate in this study during their regulatory acclimatization period. Participants were briefed before leaving France and were informed of the benefits and risks of the investigation prior to giving their written consent in accordance with the Declaration of Helsinki. This study was performed at the request of the French Forces in United Arab Emirates, approved by the scientific leadership of the French Armed Forces Biomedical Research Institute, under the direction of the French Inter-Armies Medical Center of the military city of Zayed near Abu Dhabi. All participants were found to be healthy by military physicians. The study took place in May 2016 and the participants did not participate in a mission (in France or elsewhere) where the climate could be considered hot (dry or humid) in the previous 12 months. Participant characteristics are displayed in Table 1. Each regiment was familiar with the Cooper 12-min run test (Cooper, 1968; a test routinely used by the French Army to annually assess the level of aerobic fitness). The last test was performed in the month before departure to United Arab Emirates in a temperate environment (15–20°C). Indeed, this is one of the most accurate field tests for aerobic fitness (Grant et al., 1995). Maximal oxygen uptake (VO_2max_) and speed at VO_2max_ can be estimated from the test results. In this study, running intensities were calculated from the estimated speed at VO_2max._

**Table 1:** Participant characteristics.

	**No Training (*n* = 29)**	**Training (*n* = 29)**
Age (y)	24.3 ± 3.6	24.3 ± 3.9
Height (cm)	178 ± 6	177 ± 7
Weight (kg)	75.6 ± 7.4	74.0 ± 9.1
Body mass index (kg.m^−2^)	24.0 ± 1.9	23.6 ± 1.9
12-min Cooper performance (m)	2941 ± 155	2845 ± 177
Speed at VO_2max_ (km.h^−1^)^a^	15.3 ± 1.00	14.9 ± 1.1
VO_2max_ (ml.min^−1^.kg^−1^)^a^	54.0 ± 3.5	51.9 ± 3.9

*Mean ± SD. VO_2max_ = maximal oxygen uptake*.

a *These values have been deduced from the performance of the Cooper 12-min run test (Cooper, 1968)*.

### Procedures

**Figure 1:**
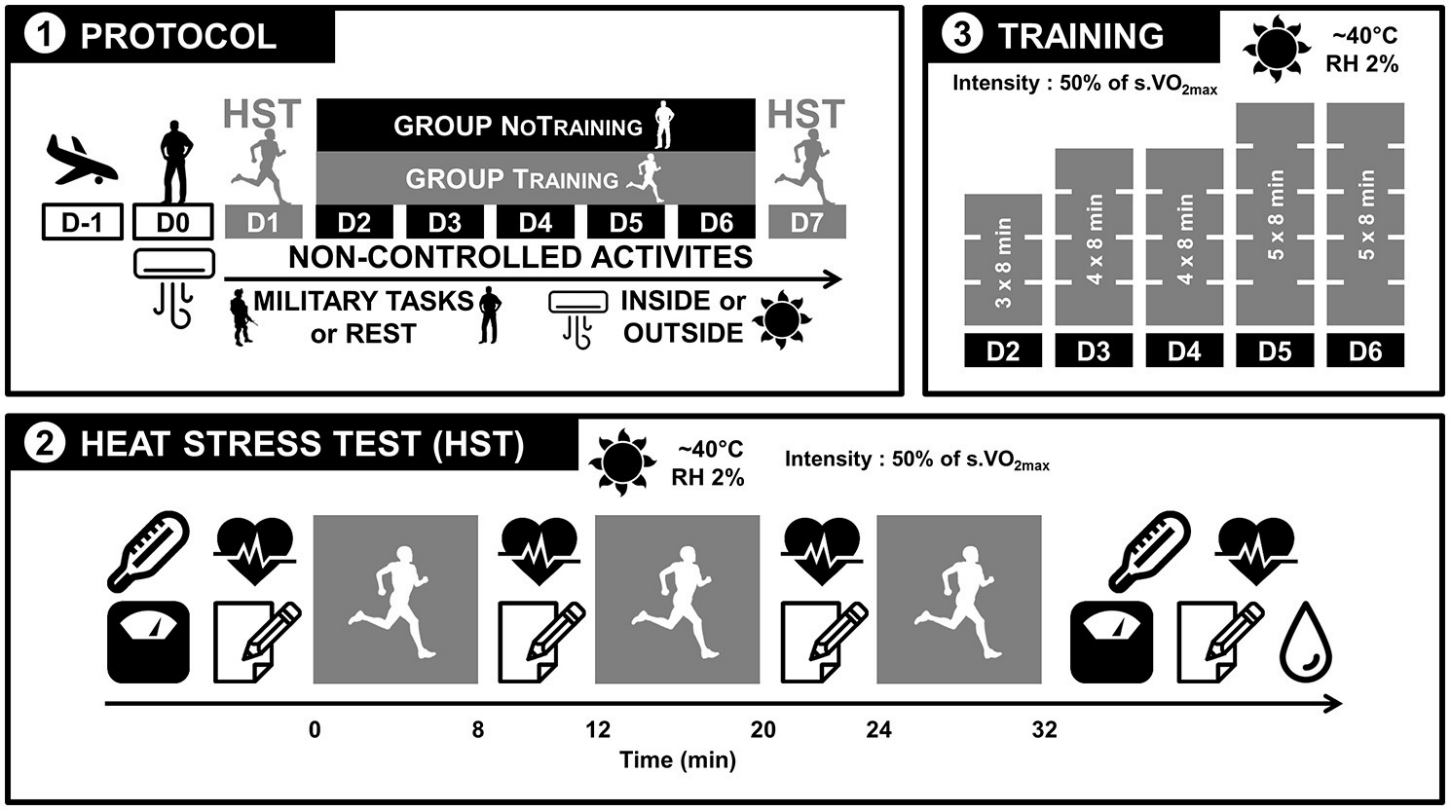
Protocol description. (1) The day after arrival, participants remained at rest in air-conditioned buildings. On D1 and D7, they performed a heat stress test (HST). Between D2 and D6, they either trained (Training [T] group) or did not (No Training [NT] group). From D1 to D7, all participants performed military tasks, mostly outdoors. These activities were not controlled. (2) The HST test consisted of three 8 min periods of running at 50% of the speed at VO_2max_ with 4 min recovery periods in between. Rectal temperature, nude and dry body mass, heart rate, and thermal discomfort were assessed before and after the HST test. During the recovery periods, heart rate, thermal discomfort, and rates of perceived exertion were measured. At the end of the tests, the sweat of some participants was recovered from sweat collectors for sweat osmolality measurements. (3) Training for the T group consisted of 3, 4, or 5 sets of 8-min periods running at 50% of the speed at VO_2max_ with 4-min recovery periods in between.

Participants stayed in air conditioned spaces for the entire day after their arrival on site by military airplane. Two days (D1) after their arrival, the participants performed a HST consisting of three 8-min runs outdoors at 50% of their estimated speed at VO_2max_. Rectal temperature, nude and dry body mass, and HR were measured and thermal discomfort assessed before and after exercise. Sweat osmolality was measured on collected sweat and the RPE assessed at the end of exercise. The sessions took place during the day when the dry temperature was between 40 and 45°C. After this test, the participants were separated into two groups: the no-training group (NT; *n* = 30) and the training group (T; *n* = 30). Each group contained the same number of members of each regiment and the anthropometric and fitness level was similar across groups (Table 1). During the next 5 days (D2-6), the T group trained daily at the same intensity (50% of the speed at VO_2max_), the duration progressively increasing (32–56 min). During training sessions, participants from the NT group remained seated outside to benefit from passive heat acclimatization. All participants performed mostly outdoor military tasks during this period. The duration of military-based physical activity and heat exposure was assessed using activity schedules filled out each evening by the section sergeant. On the last day (D7), all participants performed the same HST to assess heat acclimatization-induced modifications. The procedure is displayed in Figure 1.

#### Heat stress test

On the days of the HST, participants did not perform military tasks and were encouraged to remain in air-conditioned spaces. Given the large sample (*n* = 60), it was not possible to perform the test at the same time for all participants. The test was therefore divided into four sessions (15 participants per session: two in the morning and two in the afternoon). Participants realized the HST in the same session order on D1 and D7 to minimize environmental and especially chronobiological differences between the tests. Overnight urine samples were first collected and osmolality immediately assessed using a freezing point osmometer (Osmomat 3000 basic, Gonotec, Berlin, Germany) to determine the overall hydration status of the participants and verify whether hydration recommendations that were dispensed to all participants led to the absence of a difference between groups. The mean of the two values was conserved if two consecutive measurements diverged by <5%. If it exceeded 5%, another measurement was performed and the outlier measurement was excluded to calculate the mean. Two measurements were sufficient in 95% of cases. At least 1 h after breakfast or lunch, participants entered a room located near the outdoor track to be equipped and to measure their rectal temperature and nude and dry body mass before exercise. The air-conditioning was turned off (T~30°C) to limit the temperature gradient between the inside and outside. Upon arrival, they were weighed nude and dry (they had to dry themselves with a towel and were weighed behind a screen for privacy) with a balance constructed to resist heat, humidity, and dust (Mettler Toledo ICS 425d, Greifensee, Switzerland, accurate to 20 g). They then measured their rectal temperature with electric thermometers (PX-TH 418, Pelimex, Ingwiller, France) using disposable covers and were hidden from sight behind a screen. The measurement was taken at a depth of 6 cm. Recent studies have shown that rectal temperatures were stable at depths from 7 to 16 cm (Buono et al., 2014; Miller et al., 2017). Measurements taken at a depth of 4 cm slightly underestimated core body temperature (Miller et al., 2017). We therefore consider that our measurements well-reflect those taken deeper. They were then equipped with a chest belt and a heart rate monitor wrist receptor (RC3 GPS, Polar, Kempele, Finland). A sweat collector was placed on the lower back of some participants (*n* = 12 in each group) to collect sweat with a syringe after the test. The collector was self-made and consisted of a 10-cm plastic square held against the skin with a large transparent film dressing (15 × 20 cm; Tegaderm 1628, 3M, Neuss, Germany). Sweat was thus retained in this square during exercise and could not evaporate.

The running track consisted of a 15-m wide and 200-m long asphalt road. Tables, on which thermal discomfort visual analogical scale were displayed, were placed at the first extremity of the track. Participants had to answer the question “How do you find the thermal environment?” by placing a horizontal dash on a vertical 10 cm scale in which the bottom extremity represented “comfortable” and the top extremity “very uncomfortable.” The distance in centimeters between the lower extremity and the line gave the thermal discomfort score. This scale was adapted from previous work (Guéritée and Tipton, 2015). The participants then remained in an upright position without moving for 5 min to measure resting HR (the lowest 1 min plateau was used for the mean calculation).

The intensity was fixed at 50% of the estimated speed at VO_2max_ and participants had to run three times for 8 min with 4 min of active recovery between (from 3 to 5 km.h^−1^). Participants were grouped by fitness level for each session (no more than four groups per session). Colored cones (one color for each level) were placed along the track. A military instructor whistled every 20 s and participants had to reach the next cone by this signal. The distances between the cones were calculated to impose a speed corresponding to the desired intensity. The cones were placed such that each 8-min set finished at the same place for all group levels to facilitate post-exercise measurements. No participant failed to respect the running speed. The HR at the end of exercise corresponded to the last 30 s mean of the final 8 min run. Thermal discomfort and the RPE were immediately assessed at the end of each 8 min run (0–10 scale; Foster et al., 2001). Only measurements after the third 8 min run are presented in this article.

At the end of exercise, following completion of the thermal discomfort and RPE scales, the HR monitors were removed and participants returned to the first room to measure their rectal temperature. Sweat was recovered from the collectors for those who were equipped and stored in 2 ml aliquots. They then had to undress and towel-off meticulously for the body mass measurements. The difference between pre- and post-exercise weight was mainly due to sweat loss as food and drink intake was forbidden during the test. Indeed, insensible water loss should also be considered. We considered these losses to be between 30 and 50 g (<7% of water loss), based on the work of Houdas and Colin (1966) and considering the participant characteristics and the duration and intensity of the HST. Given that these losses were very likely similar between groups, we considered the difference between pre- and post-exercise weight to be solely attributable to sweat loss. Post-exercise rectal temperature was measured no more than 5 min after the end of exercise. The sweat collectors were then removed and the body mass measured no more than 15 min after the end of exercise. Immediately after sweat collection, osmolality was assessed with the freezing point osmometer under the same conditions and using the same protocol as for urine samples.

Environmental conditions were measured from the beginning to the end of each test with a weather meter (Kestrel Meter 440 Heat Stress Meter, Birmingham, MI, USA) near the track at a height of 1.2 m and exposed directly to the sun. At least three punctual measurements were performed. We then calculated the mean of each component: wind speed, dry-bulb, wet-bulb, and globe thermometer, and wet-bulb globe (WBGT) temperatures.

#### Training

The training intensity was controlled using the same plan as for the HST, allowing for groups of different levels. Sessions were planned in the morning or afternoon based on the daily military schedule of each section. The first session (D2) consisted of three 8 min sets at 50% of the speed at VO_2max_. The number of sets was increased to four on D3 and D4 and to five on D5 and D6 (32–56 min, including the 4-min period of active recovery).

At the end of each day, each section sergeant recorded the schedule of the day with the supervision of an experimenter. For each hour, they had to indicate where the participants were located (outdoors or indoors, with or without air conditioning) and the activity in which they were engaged (sleep, meal, rest, static, or dynamic military task). If the activity was military, they had to record the self-perceived degree of difficulty of the task using an RPE scale.

### Statistical analyses

One participant in each group was removed from the protocol, one for fever the morning of the D1 HST (NT group) and the other for a benign nose bleed during the D7 HST (T group). Thus, 29 participants in each group were considered for statistical analysis, except for sweat osmolality (*n* = 12). After ensuring that the data were normally distributed (Shapiro–Wilk test), a single mixed-model repeated-measures 2 × 2 ANOVA was used with heat acclimatization (D1 vs. D7) as the within-subject factor and trained state (NT vs. T) as the between-subject factor. *Post-hoc* comparisons of means were performed between the conditions in each group using Tukey's HSD test if an effect of acclimatization or an interaction between acclimatization and training was significant. Data in the text are presented as the mean ± *SD*. Significance was defined as *p* < 0.05. Analyses were performed using STATISTICA software (v10, Statsoft, Tulsa, OK, USA).

## Results

### Environmental conditions

Meteorological conditions were naturally not controllable. Thus, we adjusted the timing of the D7 HST sessions to minimize differences in the WBGT between D1 and D7. D7 was hotter than D1. Thus, morning sessions were performed earlier and afternoon sessions later to reduce globe thermometer temperatures to minimize differences in the WBGT. Temperatures are displayed in Table 2. The differences in the WBGT between D1 and D7 were small: between −0.9 and +1.1°C. Wind speed was, on average, slightly higher on D7 than on D1.

**Table 2:** Heat stress tests (HST) meteorological measurements.

		**HST start time**	**Dry-bulb Temperature (°C)**	**Globe thermometer temperature (°C)**	**Wet-bulb globe temperature (°C)**
Session 1	D1	10:30 a.m.	43.2	52.2	29.6
	D7	10:00 a.m.	45.3 (+2.1)	48.9 (−3.3)	29.9 (+0.3)
Session 2	D1	12:00 p.m.	43.8	55.3	30.6
	D7	11:27 a.m.	45.9 (+2.1)	55.1 (−0.2)	31.7 (+1.1)
Session 3	D1	02:45 p.m.	41.2	59.4	30.0
	D7	04:37 p.m.	42.7 (+1.5)	53.3 (−6.1)	29.6 (−0.4)
Session 4	D1	03:45 p.m.	39.7	57.6	28.8
	D7	06.00 p.m.	39.9 (+0.2)	52.1 (−5.5)	27.9 (−0.9)

*Hygrometry was similar for all sessions (12%). Values in brackets represent differences between D1 and D7 in each session*.

Fourteen participants of the T group started their training session between 9:30 and 10:00 a.m. The 15 remaining T group participants started their session at 2:00 p.m. The successive dry bulb temperatures for the morning group were 37, 37, 40, 41, and 44°C and 39, 40, 45, 47, and 44°C for the afternoon group. The relative humidity (12–16%) and solar radiation were similar during the entire training period.

### Daily schedules

Activities and heat exposure outside of the training sessions were not controlled in this study. Thus, each section was free to perform indoor or outdoor military activities, except for exercise, which was forbidden. Table 3 presents the repartition of the time spent indoors and outdoors and the nature of the tasks. The time slot between 6:00 a.m. and 11:00 p.m. was the only one considered because all participants slept in an air-conditioned room during the remaining period. Time spent outdoors performing military tasks was ~6 h per day for each group without a significant difference. The RPE for military tasks for all participants (N and NT) was rated 4.9 ± 0.8.

**Table 3:** Nature and location of daily activities.

	**No Training (*n* = 29)**	**Training (*n* = 29)**
**NATURE OF ACTIVITY**		
Rest (h.d^−1^)	10.0 ± 2.2	9.3 ± 2.1
Military tasks (h.d^−1^)	6.0 ± 2.2	5.7 ± 2.1
Training (h.d^−1^)	0.0 ± 0.0	1.0 ± 0.0
**OUTDOOR/INDOOR ACTIVITIES**		
Indoors with air conditioning (h.d^−1^)	9.0 ± 1.6	9.3 ± 1.5
Indoors without air conditioning (h.d^−1^)	0.4 ± 0.5	0.4 ± 0.5
Outdoors (h.d^−1^)	6.6 ± 2.0	6.2 ± 1.9

*Mean ± SD*.

### Rectal temperatures

**Figure 2:**
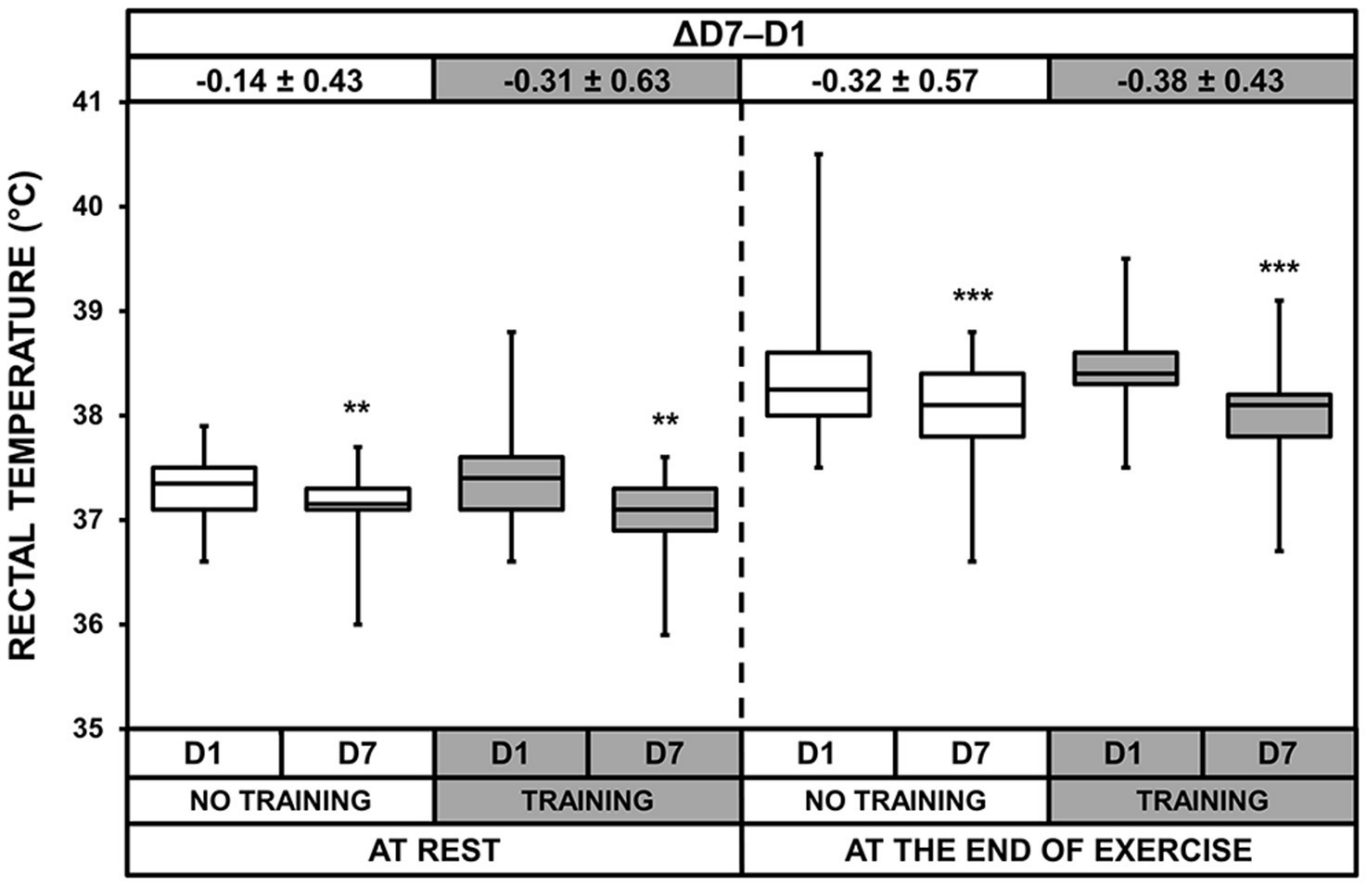
Box-and-whisker plots for rectal temperatures of the No Training and Training groups before (D1) and after (D7) heat acclimatization, at rest and at the end of exercise. The box encompasses the 25–75% quartiles, and the median is represented by the horizontal line within the box. The whiskers extend to the highest and lowest values. Values shown above the boxes, at the top of the plot, correspond to the differences (mean ± *SD*) between D7 and D1. Significantly different from D1, ^**^p < 0.01; ^***^p < 0.001.

ANOVA showed a heat acclimatization effect at rest and after exercise (*p* = 0.002 and *p* < 0.001, respectively, Figure 2) with values being lower at D7 than D1 in both groups. We found no significant acclimatization × training interaction for rectal temperatures at rest or at the end of exercise. We calculated the increase in rectal temperature during the HST (at the end of exercise minus that at rest). There was no effect of training or acclimatization for this variable (1.09 ± 0.56 vs. 0.90 ± 0.34°C for NT and 1.04 ± 0.42 vs. 0.98 ± 0.55°C for T on D1 and D7, respectively).

### Heart rate

**Figure 3:**
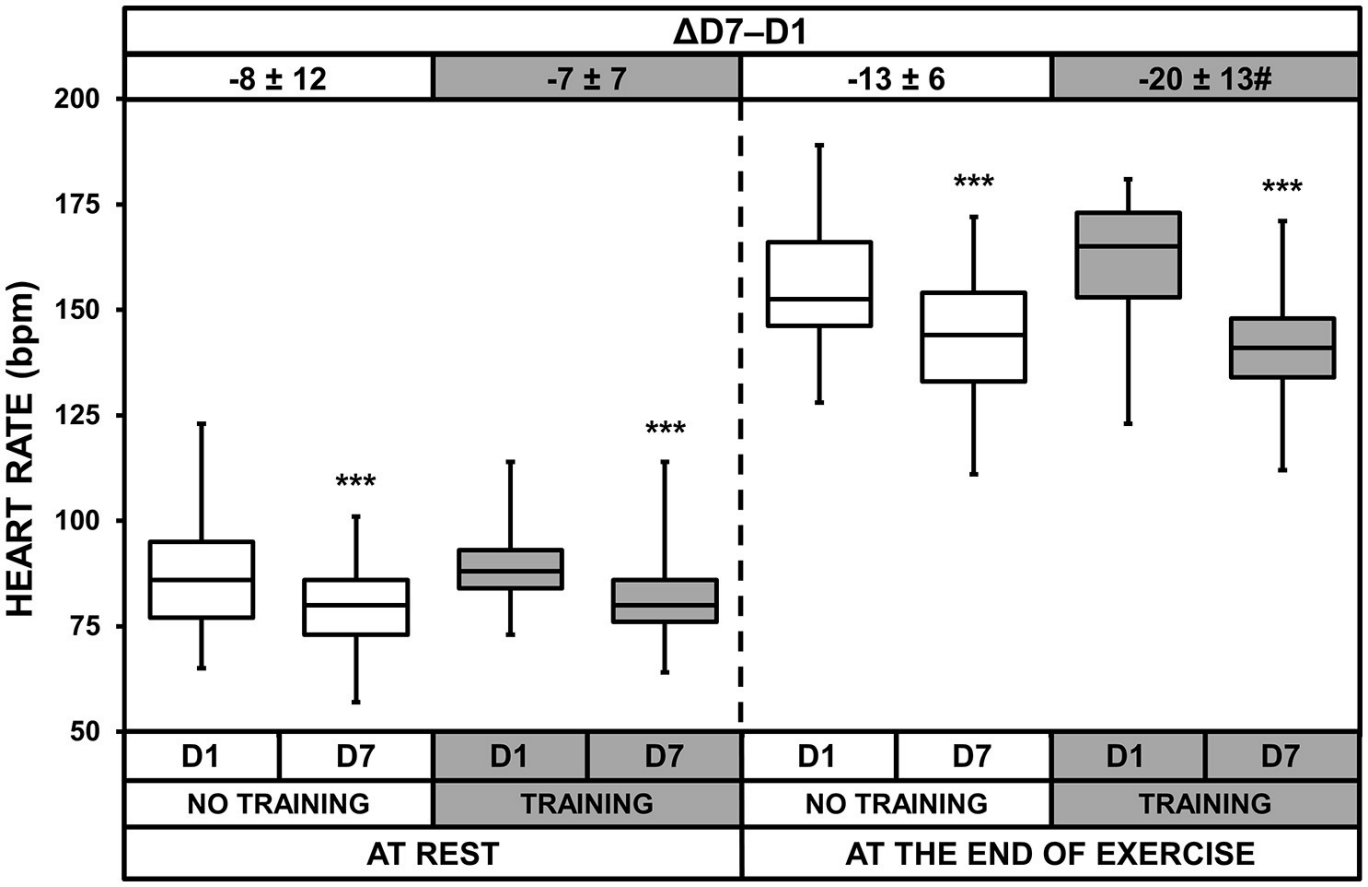
Box-and-whisker plots for heart rates of the No Training and Training groups before (D1) and after (D7) heat acclimatization, at rest and at the end of exercise. The box encompasses the 25–75% quartiles, and the median is represented by the horizontal line within the box. The whiskers extend to the highest and lowest values. Values shown above the boxes, at the top of the plot, correspond to the differences (mean ± *SD*) between D7 and D1. Significantly different from D1, ^***^p < 0.001; ΔD7-D1 significantly different from NT group, ^#^*p* < 0.05.

HRs at rest and at the end of exercise were lower on D7 than on D1 for both groups, showing an effect of heat acclimatization (*p* < 0.001 for both T and NT groups, Figure 3). Moreover, we found a significant acclimatization × training interaction for HR at the end of exercise (*p* = 0.044). *Post-hoc* analysis revealed that the decrease of HR at the end of exercise between D1 and D7 was larger in the T than NT group (*p* = 0.044).

We also calculated the increase in HR during exercise and found a heat acclimatization effect (*p* < 0.001) and an acclimatization × training interaction (*p* = 0.013). The increases were lower on D7 (65 ± 10 and 61 ± 12 bpm for NT and T, respectively) than on D1 (70 ± 13 and 73 ± 13 bpm for NT and T, respectively) and the decreases were larger in T than NT (−5 ± 9 and −12 ± 13 bpm for NT and T, respectively; *p* = 0.013).

### Sweat loss and sweat and urine osmolality

**Figure 4:**
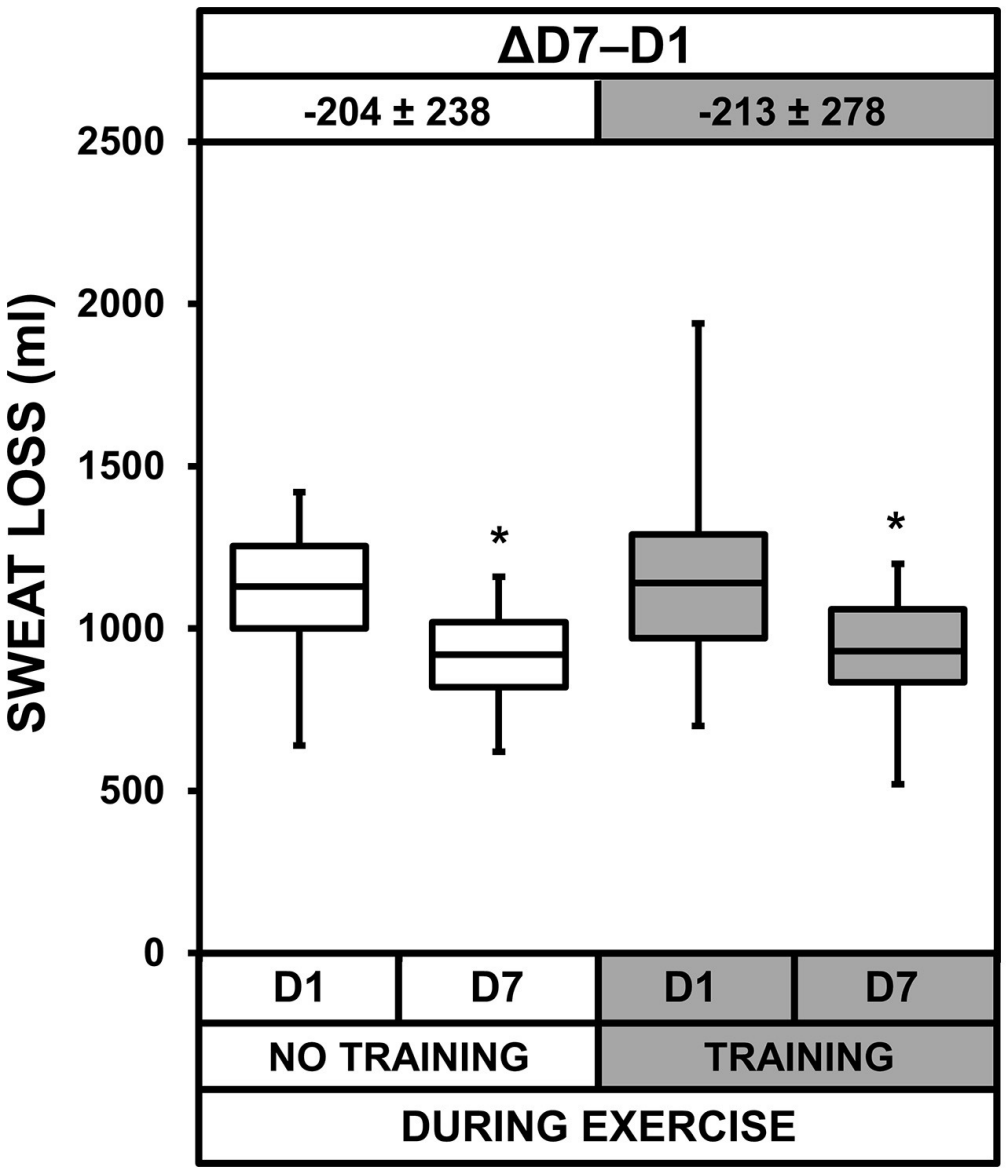
Box-and-whisker plots for sweat loss of the No Training and Training groups before (D1) and after (D7) heat acclimatization. The box encompasses the 25–75% quartiles, and the median is represented by the horizontal line within the box. The whiskers extend to the highest and lowest values. Values above the boxes, at the top of the plot, correspond to the differences (mean ± *SD*) between D7 and D1. Significantly different from D1, ^*^p < 0.05.

**Figure 5:**
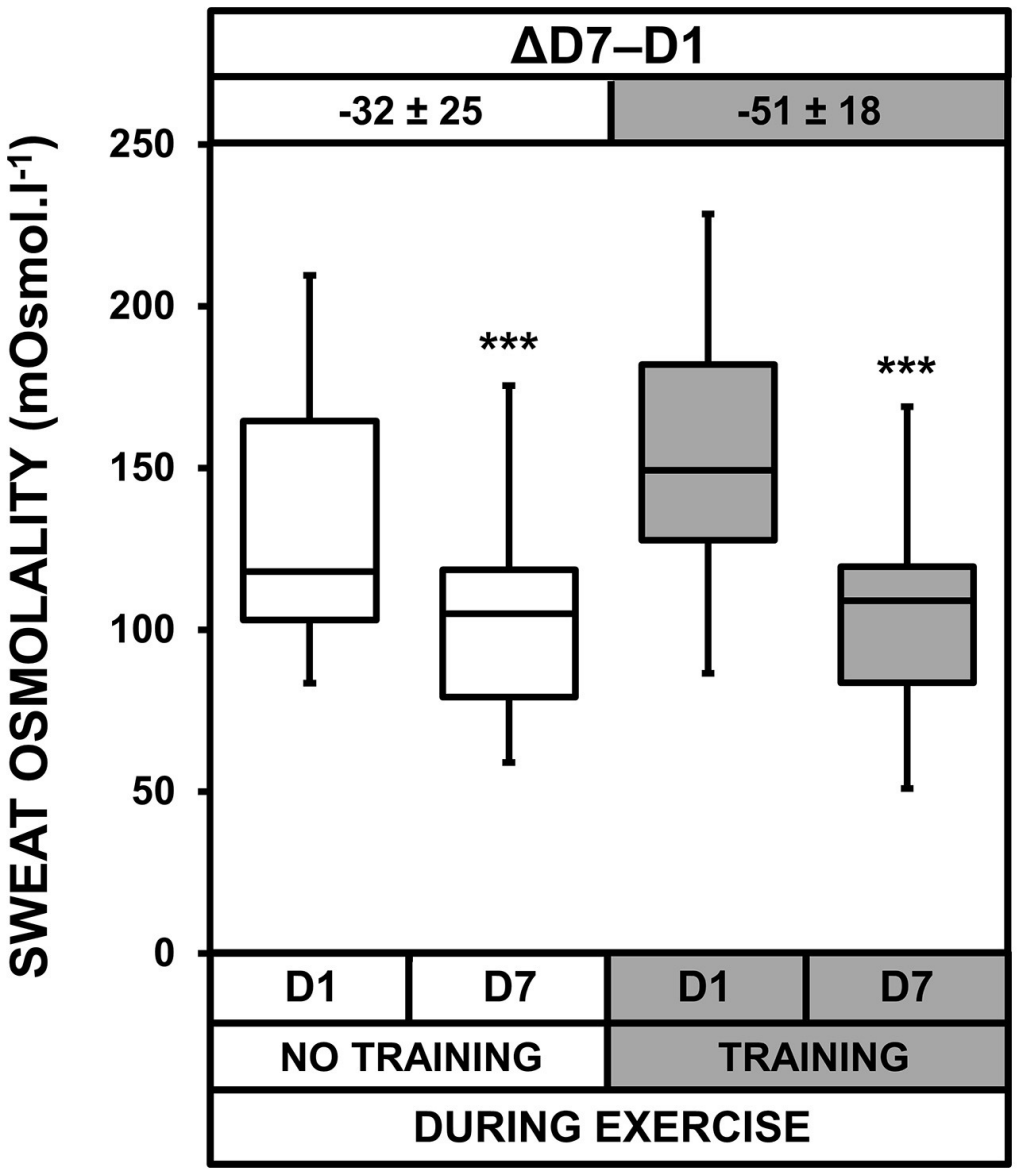
Box-and-whisker plots for sweat osmolality of the No Training and Training groups before (D1) and after (D7) heat acclimatization. The box encompasses the 25–75% quartiles, and the median is represented by the horizontal line within the box. The whiskers extend to the highest and lowest values. Values shown above the boxes, at the top of the plot, correspond to the differences (mean ± *SD*) between D7 and D1. Significantly different from D1, ^***^p < 0.001.

We observed heat acclimatization effects for sweat loss (*p* = 0.013, Figure 4), sweat osmolality (*p* < 0.001, Figure 5), and urine osmolality (*p* < 0.001). Urine osmolality increased after heat acclimatization with no difference between the two groups. Overnight urine osmolality on D7 (369 ± 155 and 397 ± 175 mOsmol.l^−1^ for NT and T, respectively) was higher than on D1 (254 ± 121 and 279 ± 93 mOsmol.l^−1^ for NT and T, respectively), resulting in an increase of 56 ± 67 and 57 ± 85 mOsmol.l^−1^ for NT and T, respectively. Sweat loss and osmolality decreased after heat acclimatization, with no difference between the two groups. The sample size for these two measurements (*n* = 12) was probably too low to detect a significant acclimatization × training interaction.

### Thermal discomfort and rates of perceived exertion

**Figure 6:**
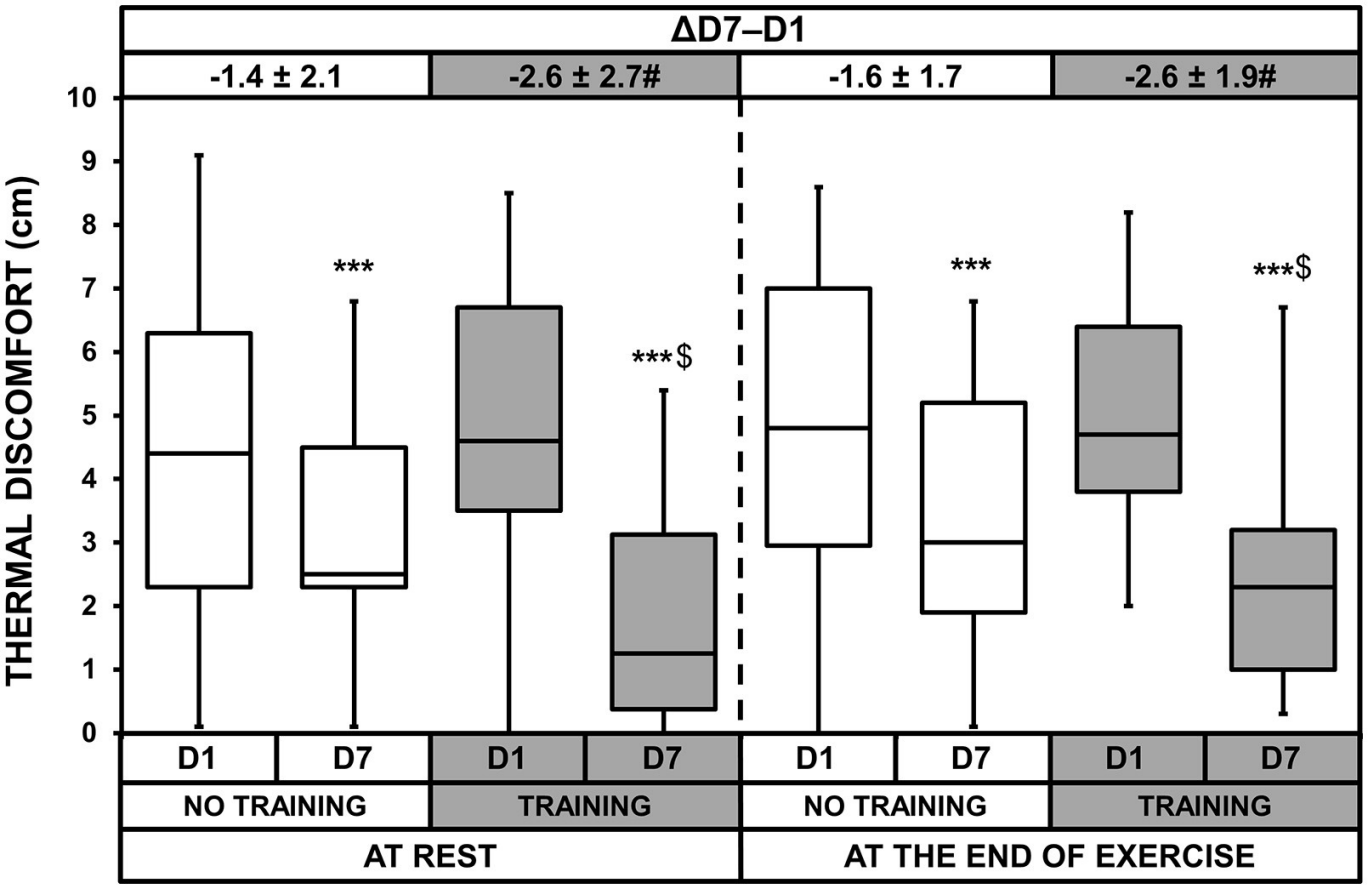
Box-and-whisker plots for thermal discomfort of the No Training and Training groups before (D1) and after (D7) heat acclimatization, at rest and at the end of exercise. The box encompasses the 25–75% quartiles, and the median is represented by the horizontal line within the box. The whiskers extend to the highest and lowest values. Values shown above the boxes, at the top of the plot, correspond to the differences (mean ± *SD*) between D7 and D1. Significantly different from D1, ^***^p < 0.001; significantly different from NT group, ^$^*p* < 0.05; ΔD7-D1 significantly different from NT group, ^#^*p* < 0.05.

**Figure 7:**
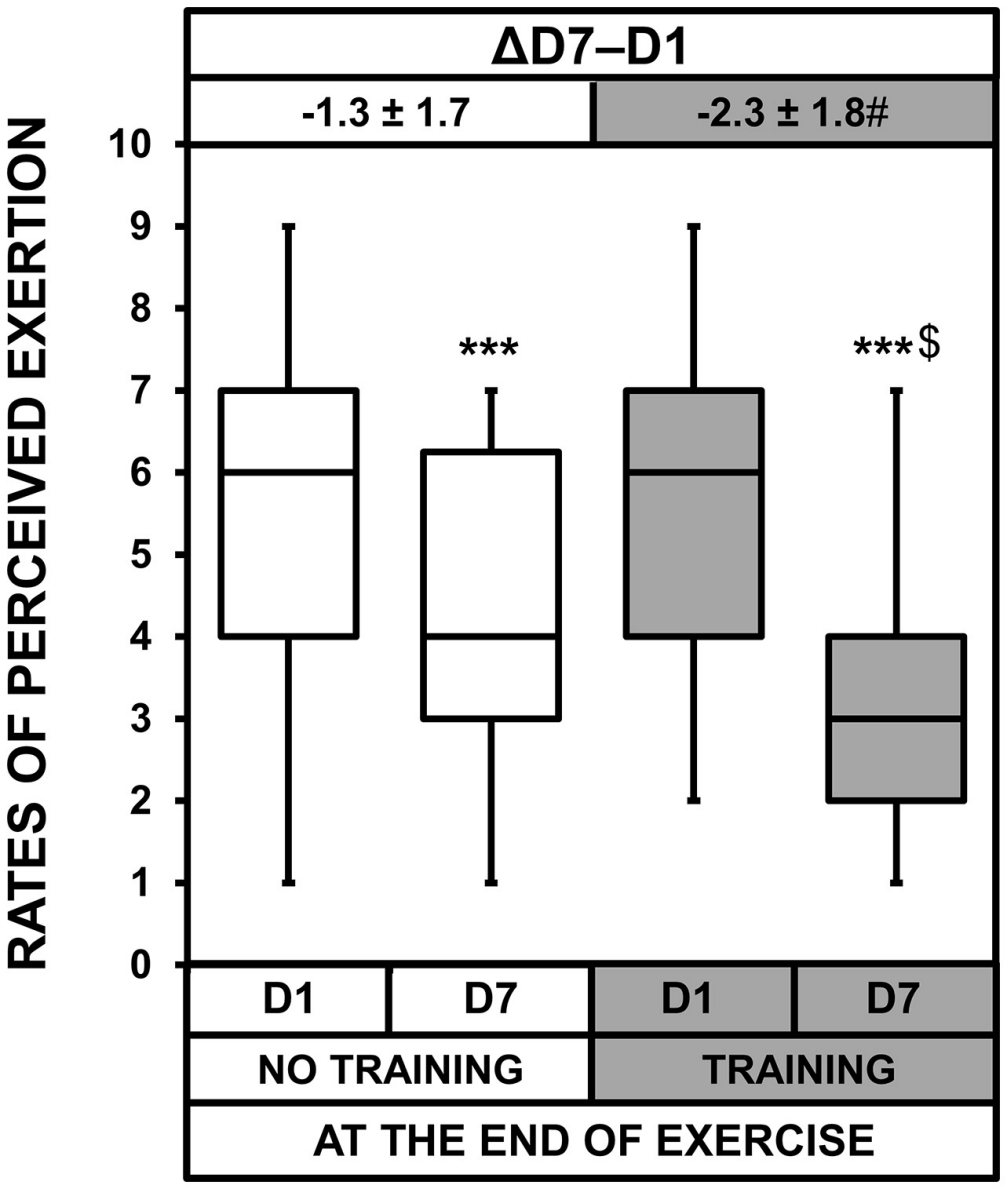
Box-and-whisker plots for the rates of perceived exertion of the No Training and Training groups before (D1) and after (D7) heat acclimatization at the end of exercise. The box encompasses the 25–75% quartiles, and the median is represented by the horizontal line within the box. The whiskers extend to the highest and lowest values. Values shown above the boxes, at the top of the plot correspond to the differences (mean ± *SD*) between D7 and D1. Significantly different from D1, ^***^p < 0.001; significantly different from NT group, ^$^*p* < 0.05; ΔD7-D1 significantly different from NT group, ^#^*p* < 0.05.

ANOVA showed a heat acclimatization effect at rest and at the end of exercise for thermal discomfort (*p* < 0.001, Figure 6). There was also a heat acclimatization × training interaction both at rest and at the end of exercise (*p* = 0.013 and *p* = 0.037, respectively). Although thermal discomfort decreased with acclimatization in both groups, these decreases were larger in the T than NT group (*p* = 0.017 and *p* = 0.037 at rest and at the end of exercise, respectively). Moreover, on D7, thermal discomfort was lower in the T than NT group at rest (*p* = 0.012) and at the end of exercise (*p* = 0.048). There was also a heat acclimatization effect (*p* < 0.001) and a heat acclimatization × training interaction (*p* = 0.035) for RPE (Figure 7). The decrease in RPE between D1 and D7 was larger in the T than NT group (*p* = 0.035). On D7, the RPE was lower in the T than NT group (*p* = 0.044).

## Discussion

A short (5 days), low-volume training program conducted during a period of heat acclimatization in a realistic context (~6 h.d^−1^ of physically stressful professional activity performed mostly outdoors) improved thermal discomfort (at rest and after exercise performed in a hot and dry environment) and post-exercise RPE, decreased HR, and trended toward diminishing sweat osmolality (*p* = 0.053, with *n* = 12) of participants after exercise, relative to those who did not receive training. These results show that short, moderate training sessions would be beneficial for personnel during missions abroad in hot and dry locales. After only 5 days of training, they would find the heat more bearable, find physical activity less difficult, and benefit from larger physiological improvements than those who did not train during the acclimatization period. However, training was ineffective in improving decreases in rectal temperature. Moreover, no symptoms of exertional heat illness were observed during the study. We did not assess these symptoms' occurrence after our intervention. However, the decrease of rectal temperatures in both groups indicates that both groups were more protected against exertional heat stroke (high core body temperature being the heat exertions).

The originality of this study lies on its being conducted in the field. Indeed, all participants continued to perform their considerable professional (military in this case) tasks, mostly outdoors, and were already acclimatized to the heat due to these activities. Thus, the training volume added to their daily routine was relatively low (between 32 and 56 min.d^−1^), but induced additional heat acclimatization.

We limited the environmental differences between D1 and D7 by adjusting the timing of the testing sessions; the maximum difference in WBGT was 1°C. Moreover, it was impossible to tightly control fitness levels of the participants and the intensity of the testing sessions. However, it was convenient and easy for soldiers to use their own Cooper 12-min run test performance to reproduce the present protocol, as it is routinely assessed in the French Army. We also gave participants practical advice concerning hydration (drink more when urine is too dark according to a urine color chart) on HST days to limit discrepancies in hydration status. There was no difference between the two groups, although urine osmolality increased between D7 and D1. Then, the training sessions in the present study was shorter (32–54 min) than those (78 min) used in short-term heat acclimation protocols (Tyler et al., 2016). Mean intensity is difficult to determine because depending on studies intensities are sometimes based on a speed, slope, or power and sometimes on relative intensity. However, the training volume used in the present study was lower than the usual heat acclimation protocols. This choice was mostly determined by practical aspects: during the first days of heat acclimatization, French Army rules stipulate that training sessions should be moderate and that their duration should be progressively increased. It would have also been impossible to insert long sessions (including preparation, recovery, and shower) into schedules that were already almost full. We thus chose the highest training volume usable in this context. Finally, the inclusion of a third group that trained in temperate conditions would have made it possible to assess the specific effect of training, regardless of heat acclimatization. However, we considered the training volume to be inferior to their usual training and it was therefore unlikely to produce a positive effect on heat acclimatization.

Reviews and meta-analyses (Taylor, 2014; Périard et al., 2015; Tyler et al., 2016) provide a precise summary of the overall kinetics of heat acclimation-induced physiological changes. After 7 days, as in the present study (i.e., 2 days of HST and 5 days of training), the maximal heat acclimation-induced improvements are almost already obtained, including HR, core temperature, and thermal comfort (Périard et al., 2015). The sweat rates do not always increase by this time. According to Taylor (2014), the first adjustments during heat acclimation are of cardiovascular origin, presumably to stabilize blood volume and blood pressures, and modifications that serve and support body temperature regulation, which interact in part with blood pressure regulation during a second phase. It is therefore not surprising that plasma volume and HR are the first outcomes to be modified and the sweat rate the last. The comparison of published work with the present study may have limitations (differences in the type of acclimation, population, and volume of training). However, the kinetics of published studies (Périard et al., 2015; Tyler et al., 2016) were almost perfectly respected: HR and rectal temperatures decreased after only 7 days (between −7.6 and −11.8% and between −0.36 and −0.97%, in the NT and T groups, respectively) and thermal discomfort greatly improved (between 15.5 and 54.3%) for all participants. Sweat loss decreased slightly (−15.5%) after 7 days, suggesting that the sweat glands were not yet able to produce more sweat in the conditions of the present study. The rectal temperature of participants decreased, despite the participants not being able to dissipate more heat through sweating/evaporation. Heat loss may be ensured through convection. Indeed, the body core temperature threshold for the onset of cutaneous vasodilation decreases during acclimation (Fujii et al., 2012; Périard et al., 2016). It is likely that blood flow was reoriented toward cutaneous tissues after 7 days of heat acclimatization, therefore promoting thermal exchange via convection. Thus, we observed positive modifications of three of the four classical markers of heat acclimation described by Sawka et al. (2011) and assessed in this study in both groups, indicating that heat acclimatization was not complete, but at least well-advanced.

The addition of training during this short heat acclimatization period produced supplementary effects on several physiological factors, despite its very low volume: (1) a smaller increase in HR at the end of exercise (−8 ± 7 vs. −12 ± 8 % in the NT and T groups, respectively) and (2) a likely larger decrease in sweat osmolality (−23 ± 15 vs. −33 ± 11% in the NT and T groups, respectively). Any decrease in HR during exercise during heat acclimation is primarily explained by plasma volume (PV) expansion (Tyler et al., 2016). The kinetics of the increase in PV and decrease of HR are almost perfectly symmetrical (Périard et al., 2015). In addition, hypervolemia increases stroke volume (SV; Tyler et al., 2016) and, given the cardiac output (CO) formula (CO = SV × HR), HR is lower for the same CO. According to Tyler et al. (2016), “A maintenance of cardiac output and arterial pressure despite reductions in HR due to compensatory increases in SV” reflects cardiovascular stability. We were unable to measure PV or stroke volume in this study. However, it is possible that PV expansion was higher in the T than NT group and allowed a larger decrease in HR during exercise therefore improving cardiovascular stability. PV expansion appears to be dependent on exercise intensity during heat acclimation (Senay et al., 1980). Although, military tasks were considered to be physical activity, the training sessions were likely more intense. The other positive training-induced modification concerned sweat osmolality and therefore sweat Na^+^ and Cl^−^ concentrations that generally decrease with heat acclimation (and acclimatization) indicating an increase in the reabsorption capacity of the human eccrine sweat gland (Buono et al., 2007; Chinevere et al., 2008). There was an almost significant difference (*p* = 0.053 for the heat acclimatization × training interaction). The small sample for this variable (*n* = 12 in both groups) potentially explains why we only approached, but did not reach, significance. It is highly likely that the training-induced decrease in sweat osmolality would have been significant with more participants and/or time. We hypothesize that inducing repeated and major sweat loss (during training sessions in the T group) conditions eccrine sweat glands to dilute sweat. Rectal temperatures at rest and during exercise were not additionally improved by training. This may indicate that physical exercise during this short heat acclimatization period did not confer better protection against exertional heat stroke, as uncompensable hyperthermia is responsible for neurological disorders and multi-organ breakdown (Epstein and Roberts, 2011). However, improvement in cardiac stability and the larger NaCl reabsorption observed in the T group theoretically confers a better protection against other heat illnesses such as heat exhaustion and syncope (Yamazaki, 2013). A follow-up of clinical signs after our training intervention would have been very informative. Regarding rectal temperature, our observations lead to several hypotheses: (1) the habitual military activities in the whole sample were sufficient to induce close to maximal heat acclimatization, (2) the training volume was not high enough to improve hyperthermia reductions and (3) the period of acclimatization was not long enough to observe differences between the T and NT groups. Literature (Henane et al., 1977; Houmard et al., 1990; Gibson et al., 2015; Périard et al., 2015; Racinais et al., 2015) strongly suggests that hypotheses 2 and 3 are very unlikely. Indeed, the choice of training volume appears to be irrelevant concerning the amplitude of heat acclimatization (Henane et al., 1977; Houmard et al., 1990; Gibson et al., 2015) and the decrease of core temperature is almost maximal after 5 days of acclimation (Périard et al., 2015; Racinais et al., 2015). To test the first hypothesis, the inclusion of a supplementary control group that did not perform military activities would have been necessary. Unfortunately, this option was not possible in the current military context.

Thermal discomfort and RPE improved in both groups, but training highly increased these improvements (−22 ± 44 vs. −49 ± 38% for thermal discomfort and −20 ± 25 vs. −38 ± 28% for RPE in the NT and T groups, respectively). Although faced with ~6 h of outdoor military tasks, the soldiers benefited from moderate training lasting <1 h.d^−1^. These results are important in an operational context. Including low-volume training upon the arrival of soldiers (or personnel from other fields) in a very hot location may help them to tolerate heat stress more rapidly, making them more comfortable and likely allowing them to perform better their professional tasks. In the present study, we did not include an HST mimicking habitual military tasks while the soldiers wore their usual outfits. However, it has been demonstrated that heat acclimation improved the ability of rally car drivers to control their vehicle during a simulation (Walker et al., 2001), the physiological and cognitive performance of soldiers during a patrol and a reconnaissance exercise (Amos et al., 2000), and the efficiency of gold miners in shoveling rocks (Schneider, 2016). Indeed, in the first half of the twentieth century, Dreosti created a heat tolerance test during which broken rocks had to be shoveled to simulate the work performed in the mines of South Africa (Dreosti, 1935, 1949). This test was used to prescribe a heat acclimatization period for each recruit. It can be argued that participants in the T group became familiarized with the HST since training sessions were based on it. It is possible that subjects in the T group learned how to anticipate this test, reducing *de facto* anxiety and therefore affecting subjective ratings. However, this effect is very difficult to assess and common to most heat acclimation studies. Indeed, facing heat during an effort should automatically familiarize test subjects, at least in part, to any other form of physical exercise performed in similar conditions. HR measured at rest, just before the post-acclimatization HST, indicates that physiological measurements were not influenced by familiarization. Thus, the lower increase in HR during the post-acclimatization HST in the T group, relative to the NT group, was solely physiological. It is therefore highly probable that the HR in the T group would increase less during any other physical activity with an intensity close to that of the HST, improving subjective ratings (thermal discomfort and RPE). This effect, along with the very likely larger decrease in sweat osmolality (*p* = 0.054), strongly suggests that tolerance to any effort in the heat (including any physical military task) would be increased in the T group. Exercise performance in the heat may improve through attenuated thermal discomfort (Cheung, 2010), although this remains to be confirmed in this context. We therefore strongly suggest that it may confer an advantage in terms of maintaining intensity and productivity.

Training intensities must be individualized to have a maximum effect. It is likely that running in a large group would reduce the effects of training of the more fit individuals. However, as long as exercise sessions produce a substantial elevation in sweat rate and core temperature (Henane et al., 1977; Périard et al., 2015), differences in session modality (volume, duration, or intensity) seems to have a modest effect on the quality of heat acclimation, although high-intensity exercise are suggested to be more efficient (Houmard et al., 1990; Gibson et al., 2015). The greatest concern is that of the less fit soldiers who would risk running at a too high relative intensity. These soldiers would face a significant risk of reaching their maximum HR and a harmful core temperature, potentially leading to heat-related illness, such as heat stroke. The relative discrepancy between subjective and physiological variables found in our study also shows the need for caution. A trained participant may feel larger improvements in thermal and exercise discomfort, likely increasing intensity during training sessions or operational tasks, based on these positive feelings/sensations. However, we showed that the range of physiological modifications do not necessarily match subjective results after 7 days in the context of the protocol. The core temperature at rest and during exercise decreased after 7 days of heat acclimatization, whether the participants trained or not. However, the increase of sweat loss, that allows better heat dissipation, had not yet occurred, implying that the maximum decrease in core temperature was not yet achieved by D7. It may be prudent to avoid high intensity tasks that risk causing adverse effects, such as heat strokes, after only 7 days of heat acclimatization, with or without training. It will be necessary to assess the physiological adaptions during a longer time period to verify whether training can accelerate the complete acclimatization process. After the HST on D1, the rectal temperatures of only two participants (one in each group) and the theoretical maximal HR of three participants (two in the NT group and on in the T group) reached thresholds generally used in laboratory studies; i.e., 39.5°C (McLellan et al., 1993; Brown et al., 2010) and 95% of the theoretical maximal HR (McLellan et al., 1993). However, these critical values were not accompanied by any other symptoms and the medical crew maintained them in the protocol. This however emphasizes the need to individualize training and limit the intensity and duration of exercise for the safety of soldiers.

To conclude, although performing low-volume physical training is not sufficient to influence rectal temperature at rest and during exercise, it enhances decreases in thermal discomfort, RPE, sweat osmolality, and HR at exercise in personnel on a mid or long-term mission (more than 5 days) who must be rapidly operational in areas with a very hot and dry climate. The present results clearly show that the addition of daily, short, moderate training sessions to existing professional duties, comprised of light to moderate physical activities under conditions of heat stress (~6 h.d^−1^), provides additional heat acclimatization in as little as 5 days. The modalities of the training sessions should be respected: (1) 30–50 min.d^−1^ of progressively increasing duration, (2) at a moderate intensity (50% of VO_2max_) and (3) taking care to rehydrate after sessions. We showed that such training is safe under these conditions. If medical surveillance is unavailable, especially if participant(s) is (are) not at least in moderately good physical condition, sessions should be stopped at the first appearance of early heat illness symptoms (headaches, nausea, weakness or excessive fatigue, dizziness, excessive thirst, muscle aches, confusion, anxiety) and body cooling should be sought (shower or bath in water under 30°C, rest in an air-conditioned space and water consumption). We do not guarantee similar results in humid conditions (>40%), although the literature tends to show that physiological changes occur more rapidly in humid than dry conditions (Nag et al., 1996; Griefahn, 1997; Périard et al., 2015).

## Ethics statement

This study was specific since it was realized in a military context. A soldier section is sometimes designed by its hierarchy to perform an experiment as long as non-invasive measurements are realized, their health is not jeopardized and as long as the intervention is strictly similar as their usual military activities. We then recruited volunteers in this section to perform experiments. Thus, no ethics committee was necessary in this context to start that kind of experiment.

## Author contributions

KC, NK, MR, LJ and AM designed the study. PT, JB, SB, BL, MR, LJ, and AM performed the research. KC conducted the statistical analyses and drafted the initial manuscript. All authors reviewed and revised the manuscript, approved the final manuscript as submitted and agree to be accountable for all aspects of the work.

### Conflict of interest statement

The authors declare that the research was conducted in the absence of any commercial or financial relationships that could be construed as a potential conflict of interest.
